# Margination of Stiffened Red Blood Cells Regulated By Vessel Geometry

**DOI:** 10.1038/s41598-017-15524-0

**Published:** 2017-11-10

**Authors:** Yuanyuan Chen, Donghai Li, Yongjian Li, Jiandi Wan, Jiang Li, Haosheng Chen

**Affiliations:** 1 State Key Laboratory of Tribology, Tsinghua University, Beijing, 100084 China; 2 Department of Microsystem Engineering, Rochester Institute of Technology, Rochester, NY 14623 USA; 3 School of Mechanical Engineering, University of Science and Technology Beijing, Beijing, 100083 China

## Abstract

Margination of stiffened red blood cells has been implicated in many vascular diseases. Here, we report the margination of stiffened RBCs *in vivo*, and reveal the crucial role of the vessel geometry in the margination by calculations when the blood is seen as viscoelastic fluid. The vessel-geometry-regulated margination is then confirmed by *in vitro* experiments in microfluidic devices, and it establishes new insights to cell sorting technology and artificial blood vessel fabrication.

## Introduction

In blood vessels, red blood cells (RBCs) preferentially travel in the low-shear zone in the channel center, while white blood cells (WBCs) and platelets flow near the channel walls. This well-known phenomenon results in the formation of a depletion layer of RBCs near the wall but the margination of WBCs and platelets^1,2^. The lower deformability of WBCs and platelets is widely accepted as the main reason for the margination^3^. On the other hand, diseases of malaria, sickle cell anemia, and diabetes also cause RBCs to lose their deformability^4–6^, but whether stiffened RBCs could perform margination in blood vessels has not been reported yet. The current studies on the margination of RBCs *in vitro* have found different margination results in the circular capillaries and in the rectangular channels^7–9^. The margination is also found to be affected by the different hematocrit (Hct) in the experiments. Therefore, *in vivo* margination of stiffened RBCs needs to be performed in the real blood vessel with physiological concentration of Hct to clarify the margination of RBCs.

It’s known that cells will flow along their streamlines in the microchannel and will not perform lateral motion when their size is small enough to be omitted comparing to the channel size. But when the cell’s size cannot be omitted, it will migrate towards the center of the channel where the low-shear zone locates, and its deformability would take effect on the margination speed and the equilibrium position on the channel cross section^10,11^. Furthermore, when the fluid is viscoelastic, such as the blood with 30~45% Hct^12^, the lateral motion of the cell will be influenced by the first normal stress of the viscoelastic fluids, and the distribution of the normal stress would be affected by the vessel geometry^11,13–15^. Therefore, the geometry of the blood vessel would play an essential role in the margination of RBCs *in vivo* that would clarify the margination of cells in many vascular diseases, while the design of the channel geometry may control the margination of RBCs *in vitro* that would establish new insights to the cell sorting technology. The structure of vessel, such as the curvature and the bifurcation have been proved to be crucial to the cell margination^11,16,17^. But the cross section of the channel is usually rectangular or circular, the effect of the vessel cross section on cell margination has not been reported yet.

In this work, we conduct both *in vivo* and *in vitro* experiments to study the margination of the stiffened RBCs. The effect of vessel geometry on the margination of stiffened RBCs is illustrated while the blood is seen as a viscoelastic fluid. Our findings show previously unidentified roles of vessel geometry on RBCs margination *in vivo*, and the geometry effect has been quantitatively investigated by numerical calculations and *in vitro* experiments.

## Results

### *In vivo* RBCs margination in blood vessels with irregular geometry

**Figure 1:**
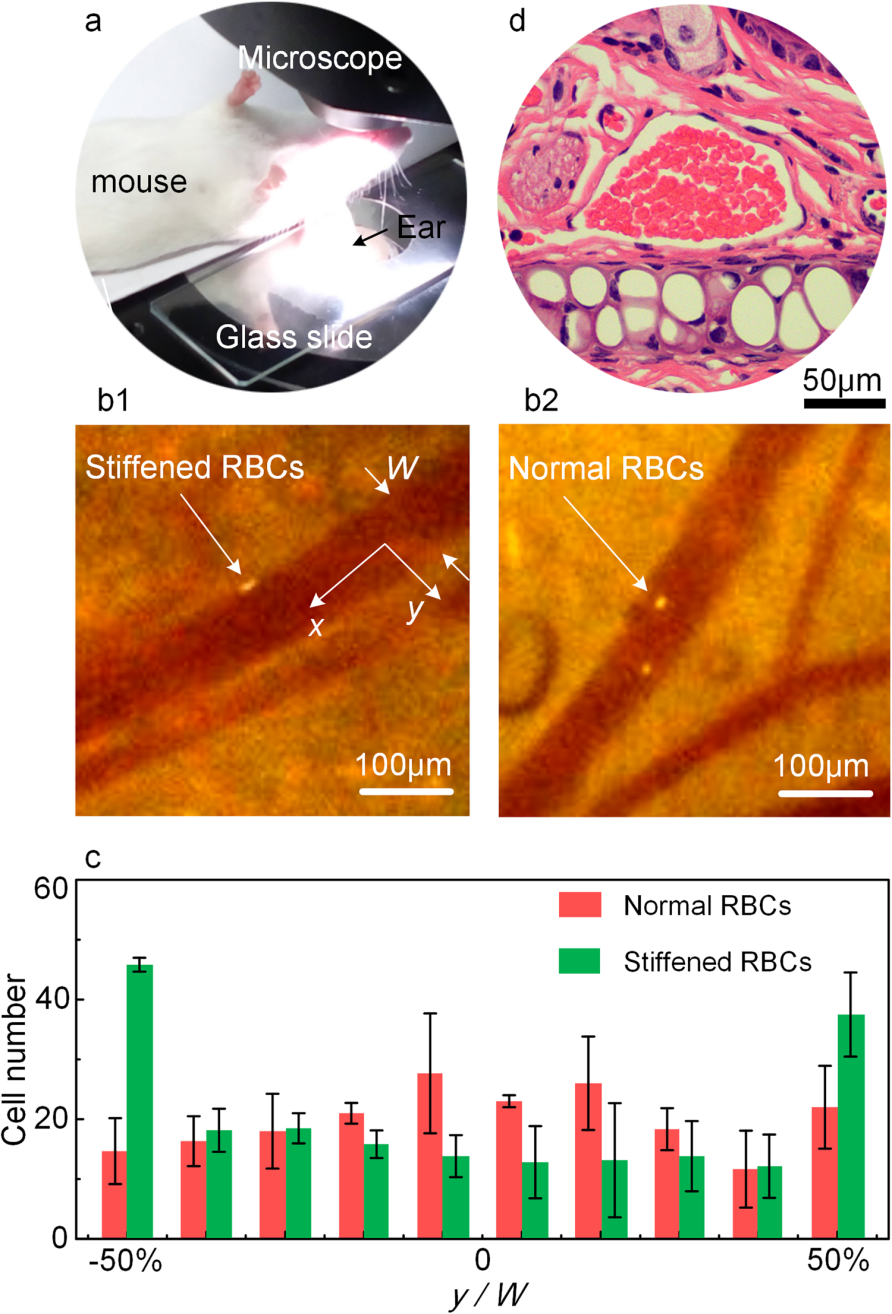
Stiffened RBCs perform margination *in vivo*. (**a**) The *in vivo* margination experiment performed in venules of a mouse under microscope. (**b1**) and (**b2**) The flowing of fluorescent stiffened and normal RBCs in mouse ear venules, respectively. The corresponding videos of the motion of the RBCs are provided in Supplementary Video-Movie 1 and Movie 2, respectively. (**c**) Distributions of stiffened and normal RBCs in mice venules. (**d**) The tissue slice to show the geometry of the venule. It is irregular and RBCs are seen inside. The white part is the cartilage tissue.

RBCs taken from healthy mice are stiffened and stained (see Methods), and then are injected into the heart of mice and enter the blood circulation. The mouse is laid on the platform of an inverted microscope (Leica DME6000 B) with its ear clinging to a glass slide as shown in Fig. 1(a). When the stiffened RBCs reach the ear, their motions inside the venules, with a diameter of 50–100 μm, are observed through a high-speed camera (Phantom M110), as shown in Fig. 1(b1,b2). The lateral position of 200 stiffened RBCs in the venules in one mouse is counted, and the statistical results from three mice are presented in Fig. 1(c). It is found that the stiffened RBCs flow near the wall of venule and margination occurs in the venules. The same experiment is also performed on normal RBCs, but their lateral distribution is homogeneous in the venule, and margination does not occur.

It is acknowledged that rigid particles would move toward the center in the circular channel where locates the minimum shear gradient^10,14,18^, while the stiffened RBCs in the venules present a different margination close to the wall. The most possible reason is considered to lie in the geometry of the venules. According to the previous studies as discussed in the introduction, the geometry of the vessel can change the direction of the normal stress to induce the margination of particles when the fluid is viscoelastic^11,13,14^. Therefore, the venules are supposed not to be circular as they are commonly recognized. To validate this, tissue slices of the venules are made to show the geometry of the cross section after the experiments (see Methods). The cross section of the venules shown in Fig. 1(d) confirms that the venule geometry is not circular but irregular. Images of more slices are provided in Supplementary Materials I. It explains why stiffened RBCs perform margination in the venules, and reveals the essential role of the vessel geometry in the motion of RBCs in blood vessels. The quantitative analysis on the relationship between the vessel geometry and the margination of stiffened RBCs are provided as following.

### The mechanism of margination regulated by vessel geometry

**Figure 2:**
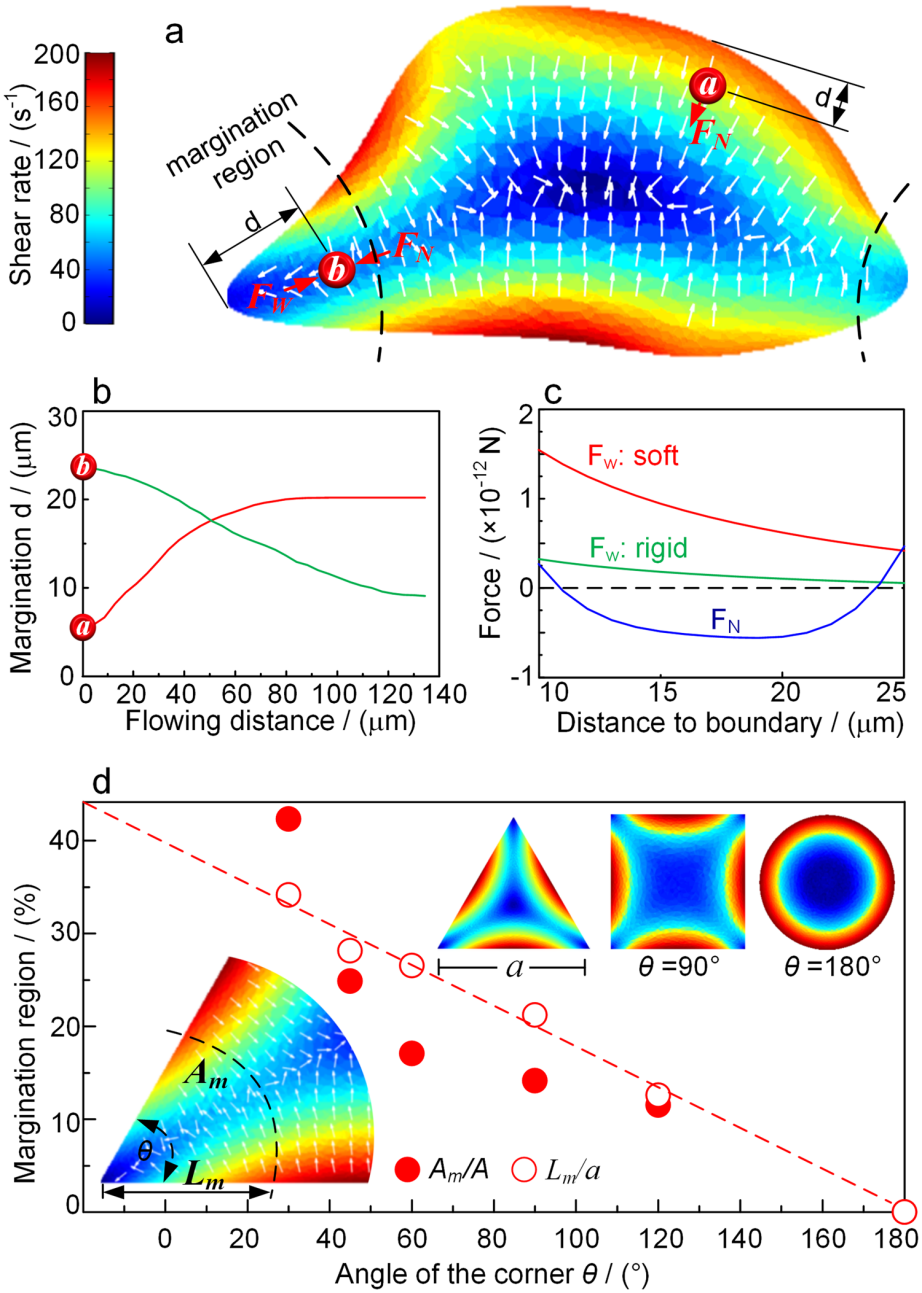
Calculations of RBCs margination in vessel and channel. (**a**) The distribution of shear rate (color) and the first normal stress (arrow) on the cross section of the blood vessel. (**b**) Trajectories of the two RBCs moving towards the center and towards the wall depending on their initial positions. (**c**) Comparison of the wall lift forces on the normal and stiffened RBCs changed with the distance to the wall as well as the first normal stress located at position of cell *b*. (**d**) Calculated margination region in different geometries represented by the angles of the corners.

The first normal stress (F_N_) is numerically calculated (see Methods) in the venule with the geometry acquired from the slice shown in Fig. 1(d). The calculated local shear rate is shown by the color in Fig. 2(a), while the gradient of the shear rate is shown by the arrows. Since the first normal stress^13,19^ could be expressed as:

1 $${F}_{N} \sim \mu \lambda \nabla {\dot{\gamma }}^{2}$$

where *λ* is the relaxation time of the solution, *μ* is viscosity, and $\dot{\gamma }$ is local shear rate, the direction of the normal stress is the same as the gradient of the shear rate $\nabla \dot{\gamma }$. The direction of the gradient of the shear rate in the figure is seen to be affected by the geometry of the vessel cross section, and the first normal stress has different directions depending on the position at the channel. For example, when the RBC is near the boundary with obtuse angles, as shown by the red cell “*a*” in the figure, the normal stress is pointing to the center and it will push the cell towards the center of the channel. But when the RBC is near the corner with acute angles, as shown by the red cell “*b*” in the figure, it will move towards the corner since the normal stress here is pointing to the wall. The trajectories of the RBCs at the two places along the flowing distance are calculated and shown in Fig. 2(b).

As a stiffened RBC approaches the corner under the first normal stress, the wall lift force, F_w_, is induced by the hydrodynamic interaction between a RBC and the wall^20^.

2 $${F}_{{\rm{w}}}=f(1-v){R}^{3}\dot{\gamma }/(6\pi {d}^{2})$$

where *R* is the radius of the cell, *d* is the distance to the boundary, the deformability of RBC is presented by *f*(1 − *v*), which is a dimensionless function to distinguish the deformability of cells, *v* is the reduced volume of the cells, which is defined as the ratio of the volume of the vesicle relative to the volume of the sphere with the same surface. For example, for stiffen leukocyte *v* = 1.0 and *f*(1 − *v*)* = *0.2, for deformable RBC *v* = 0.7 and *f*(1 − *v*) = 1.5^21,22^. Therefore, the normal RBCs have a relatively larger wall lift force than the stiffened RBCs, as illustrated in Fig. 2(c). For a stiffened RBC, when the RBC is near the vessel wall, the direction of F_w_ and F_N_ is opposite, thus, the stiffened RBCs would have an equilibrium in the margination region and the margination occurs. While, for the normal RBC, because of the higher F_w_ near the vessel wall, it will be pushed away the wall and out of the margination region (as described in Fig. 2(d)), where the F_N_ acted on the normal RBC will change its direction to the vessel center, which is the same to the direction of F_w_, thus, the margination won’t occur for the normal RBCs.

Therefore, stiffened RBCs would perform margination when they are in the region where the normal stress is pointing to the wall. For regular geometries, the region of margination in the vessel can be calculated according to the distribution of the normal stress near the corner, as shown by the insert schematics in Fig. 2(d), where the length ratio *L*_*m*_ */a* and area ratio *A*_*m*_ */A* represent the margination region with the bottom angles changing from 30 to 180°. *a* and *A* are the size and area of the cross section, the subscript *m* represents the margination region. The calculated margination area decreases with the increase of angles, and the decreasing gradient is Δ*L*_*m*_ */a* ≈ 2%/10° according to the fitting line. The margination for circular is zero because all the gradient is pointing to the center of the channel, and no margination would occur. This calculation indicates that the margination of cells can be controlled through the geometry design of the microchannel cross section.

### *In vitro* margination of stiffened RBCs

**Figure 3:**
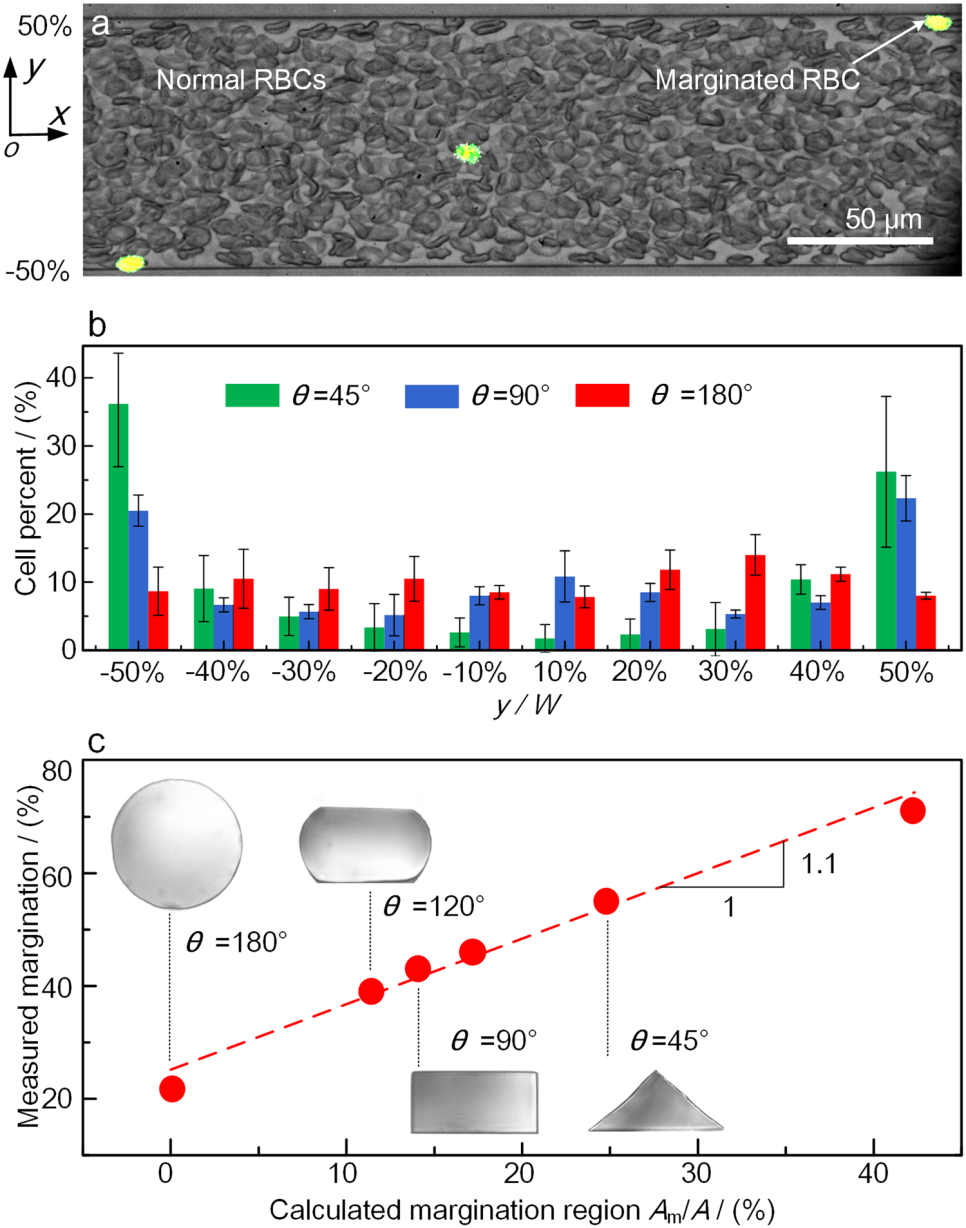
*In vitro* experiment on the margination of stiffened RBCs. (**a**) The fluorescent stiffened RBCs flowing with 30%Hct normal RBCs in a rectangular microchannel. The image of the fluorescent stiffened RBCs is superimposed with the image of the RBCs in the bright-field. (**b**) The results of stiffened RBCs margination in triangle, rectangular and circular channels. (**c**) The measured margination compared to the calculated results. The inserted images show the cross section of the channel with different corner angles, and the size of the channel is 100 μm.

*In vitro* experiments are performed to validate the calculated effect of vessel geometry on the margination of RBCs in microchannels with the geometry angles from 30–180° (see Methods). Stiffened RBCs are injected into the microchannels with 30%Hct blood. The fluorescent stiffened RBCs is seen to move laterally to the sides of a rectangular channel to perform the margination as they travel along the flow direction in the channel, as shown in Fig. 3(a). It is noted here that for triangle, rectangular and hexagonal channels (θ = 30, 45, 60, 90, 120°), the focusing plane of the microscope is on the channel bottom to observe the margination at the corners, while for circular channel (θ = 180°), the focusing plane is at the middle of the channel.

Stiffened RBCs were suspended in 30%Hct RBCs to form the 30%Hct sample (see Methods), and the lateral positions of 200 stiffened RBCs are counted and the distribution in three kinds of channels with *θ* = 45°, 90° and 180° are compared in Fig. 3(b). In the rectangular channel, the number of the stiffened RBCs on both sides of the channel is obviously higher and the margination happens in the channel. In the triangle channel, the margination phenomenon is more obvious, and larger percentage of stiffened RBCs perform margination to the bottom corners. However, stiffened RBCs are not accumulated in the center of the circular channel as predicted. The reason lies in the fact that the focusing plane is on the middle of the channel, and the fluorescent RBCs may be concealed by the RBCs below the focusing plane, which are not counted in the distribution since they cannot be determined to be on the focusing plane or not. The distribution of the cells at the center of the channel will be provided in the Discussion section, while the distribution presented here at least illustrates that margination does not occur in the circular channel.

To quantify the margination of stiffened RBCs in different channels, the percentage of the RBCs on both sides of the channel are counted and compared to the calculated margination area (*A*_*m*_ */A*) % as shown in Fig. 3(c). The linear fitting line in the figure has a slope ~1, which validates the numerical results on the margination of stiffened RBCs in channels with different geometries. It confirms the essential role of the vessel geometry in regulating the stiffened RBCs’ margination both *in vivo* and in *vitro*, and illustrates the controlled margination of cells through the design of vessel geometry.

## Discussion and Conclusion

This *in vivo* study reveals that RBCs could perform margination in blood vessels which is usually thought to happen on WBCs and platelets in blood vessels. The finding of the irregular geometry of the venules clarifies the essential role of the geometry on the margination of stiffened RBCs in viscoelastic blood. It is true that most arteries and larger veins are circular because the blood pressure inside is usually high and they have muscle layers to keep their shape. However, the blood pressure in small venules is not high, and the endothelial layer around the venules would form an irregular shape as we found in the experiments. The corners on the irregular cross section provide a necessary condition for the first normal stress to change its direction to allow stiffened RBCs to perform margination. This finding may also help the simulation of the blood flows in the vessels to find more interesting physiology behaviors of the blood.

**Figure 4:**
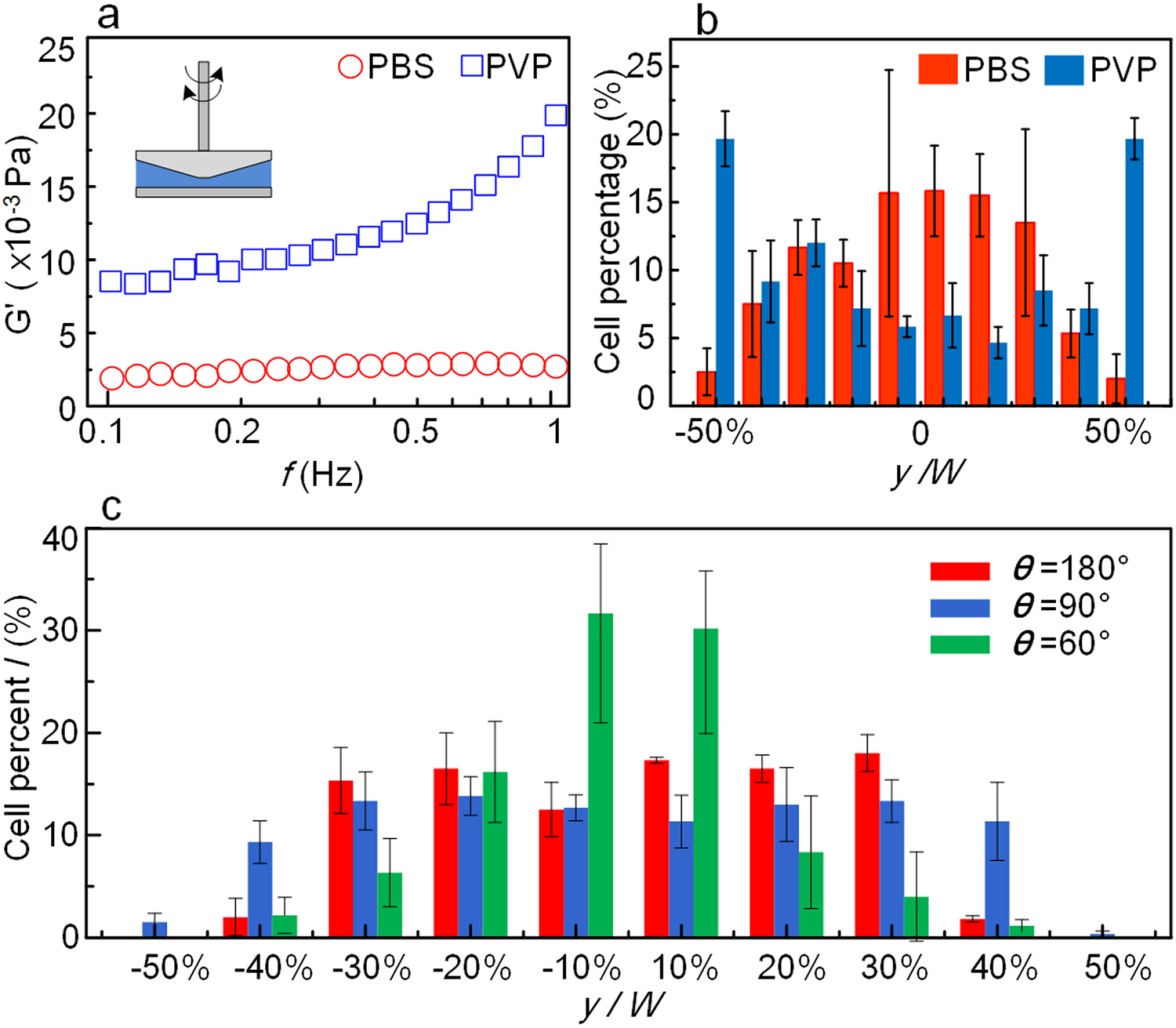
Viscoelastic effect of the fluid on the margination and the distribution of stiffened RBCs in the center of the channel. (**a**) The measured viscoelasticity of PBS, PVP solution. (**b**) The margination of stiffened RBCs in PBS and PVP solutions in rectangular channels. (**c**) The distribution of the stiffened RBCs at the middle focusing plane of the three different channels.

The viscoelasticity of the blood is another necessary condition for the margination, and has been reported in previous work^23^, and the suspension of the RBCs is one of the important reason to generate the viscoelasticity of blood. In the experiment, the washed packed RBCs were added into PBS solution to simulate the whole blood which is a common method in many experiments, and the viscoelasticity is the whole effect of the RBCs suspension. It would have no effect in the Newtonian fluid which is validated by the experiment using phosphate-buffered saline (PBS) and polyzvinyl pyrrolidone (PVP) solutions in rectangular channel (see Methods). The viscoelasticity of the solution is evaluated by the storage modulus (G’) measured by a rotation rheometer, as shown in Fig. 4(a). According to the storage modulus of PBS solution, it can be seen as Newtonian fluid, and the margination does not appear in the PBS solution as shown in Fig. 4(b). The same experiment is also performed in the PVP solution, which has an obvious viscoelasticity as shown in Fig. 4(a), and the stiffened RBCs perform obvious margination in the PVP solution as shown in Fig. 4(b). Especially, the channel height used in Fig. 4(b) is 10 μm, and this lower channel allows to see the margination of the stiffened cells on both the top and bottom wall of the channel to illustrate a more obvious margination effect compared to that in the PBS solution. The experiment results of margination in 40 μm height rectangular channel was also provided in the Supplementary Materials II. This experiment confirms that the cell margination could be realized by the fluid viscoelasticity, even without the collisions between cells. In this study, the blood is treated as viscoelasticity fluid^24–26^, and the flow patterns of the margination region depending on the geometry of the cross section can be calculated, which illustrates the direction change of the normal stress in and out of the margination region. It is true that the collision of cells may play an important role in the cell motion in the vessel^7,27–31^, but here the role of the cross section in the flow pattern near the corner is difficult to be illustrated by the collision theory. On the other hand, it has been reported that the collision effect could be ignored when the hematocrit is high, for example, higher than 30%^32,33^, while the fluid viscoelasticity effect become more important to the cell margination under this condition. Therefore, compare to the collision theory, viscoelasticity fluid model is preferred to be used here to explain the geometry effect on the stiffened cells margination, and it may help us to understand the effect of the local flow pattern near the corner of the channel.

According to the calculations on the distribution of normal stress, there is an equilibrium position in the center of the vessel besides at the corners. But in the experiment, the center equilibrium is not illustrated either because the focusing planes are on the bottom of the channels or because the fluorescent cells are concealed by the normal RBCs in the circular channel. Here we move the focusing plane to the middle of the channels while transparent PVP solution is used. The margination of stiffened cells towards the center of the channel then appears as shown in Fig. 4(c), where the percentage of the cells in the center is obviously higher than that on both sides of the channel. Especially, the channels used here are the same with Fig. 3, and the experimental results in rectangular channel illustrated that 40 μm is high enough for the microscope to distinguish the center and bottom of the channel.

In summary, we report the *in vivo* margination of stiffened RBCs in blood vessels with physiological concentration of RBCs and reveal the regulatory role of vessel geometry, which has been confirmed by both theoretical calculations and *in vitro* microfluidic experiments. This investigation on RBCs margination may not only offer new insights to the RBCs behaviors in real blood vessels when they are infected by malaria or diabetes, but also help to design microfluidic devices for the cell sorting and to fabricate artificial vessels for potential medical applications.

## Methods

### RBCs preparation and *in vivo* experiment set up

The RBCs used *in vivo* experiment were taken from the tail of mice (strain: CD1; age: 6week; level: SPF). Mice were anesthetized by avertin (1.2% Tribromoethanol) with the dosage of 200 μl/kg on enterocoelia. After washing, the RBCs were firstly stained by fluorescein isothiocyanate isomer I (Sigma-Aldrich). Fluorescein isothiocyanate (1 mg) was mixed with 1 ml phosphate-buffer saline (PBS, 1X without Ca^2+^ and Mg^2+^, Hyclone), and the buffer was centrifuged at 3000 r/min for 5 minutes. Then, the clear stain solution was added into 10 μl prepared RBCs. The solution was incubated in water bath at 37 °C for 3 hours. After incubation, the solution was centrifuged and washed using PBS 5 times to remove the stain. Half of the RBCs were treated by diamide (200 μM, Sigma-Aldrich) for 10 minutes to become stiffened. The elastic modulus of the stiffened cells was measured using an atomic force microscope (AFM) (see Supplementary Materials III). The stiffened RBCs were added into PBS with the concentration of 0.3%v/v and 200 μl of the suspension was injected into the heart of mice.

The study protocol was approved by animal care and use committee of Tsinghua University (AP#16-ZY2). The mice received humane care according to the laboratory animal management regulations. All the researchers associated with this research project have received the appropriate training and have the qualification of animal experiment. All activities associated with this research project were performed in accordance with laboratory animal management regulations.

### Venules slice preparation

To keep the shape of the blood vessels before it was made into slices, the piece of ear was moved into the formalin (37.0–40.0%, Xilong Scientific) immediately to stabilize the ear vessel tissue after the flowing observation experiment. After the sequences of water scrubbing, dehydration, transparency, waxdip, embedding, slice up and slice fixation, the venule slice was stained. The stained slice was dehydrated, sealed and finally fabricated. The procedures were performed according to the standard method as described in previous studies^34^.

### Calculations of the first normal stress

Comsol Multiphysics software is used to solve the mass conservation and the Navier-Stokes equations to get the shear rate on the cross section of the channels. The numerical details including fluid properties, algorithm, boundary conditions, initial conditions are provided in Supplementary Materials IV. After the distribution of the shear rate is acquired, the first normal stress is calculated according to equation (1), where the gradient of the shear rate is calculated using Matlab software, and the F_N_ shown in the figure is normalized by its maximum value.

### Microchannel

Microchannel with rectangular cross section was made of PDMS using standard lithographic method^35^, and the width for all rectangular microchannel is 100 μm(W), while the height is 40 μm in Fig. 3 and 4(c) for geometry controlled experiment, 10 μm in Fig. 4(b) for viscoelasticity controlled experiment. Microchannel with circular and irregular cross sections were fabricated using copper wires, and the width of the wires is 100 μm (W). The wires were fixed on the middle of petri dish with a distance of 1mm to the petri dish bottom. Then PDMS was poured in until the fluid immerse the wires. After air exhausting and baking, the wires were pulled out to form the channels with circular and irregular geometries in the PDMS devices. Microchannel with triangular cross section was made from metal mold fabricated by mechanical processing. The channel length is 3 cm for all channels.

### Viscoelastic solutions for *in vitro* experiment

Three samples were injected into the microchannel at a mean flow rate of 1 mm/s (the corresponding flow rate in each channel is: 0.47 μl/min for circular channel, 0.24 μl/min for rectangular channel with height of 40 μm, 0.06 μl/min for rectangular channel with height of 10 μm, 0.36 μl/min for irregular channel, 0.26 μl/min for 60° triangle, 0.15 μl/min for 45° triangle, 0.09 μl/min for 30° triangle), respectively, which is consisted with the mean velocity 0.61 ± 0.17 mm/s of RBCs flowing in the venules in mice ear obtained from *in vivo* experiment.

*30%Hct sample:* The washed RBCs suspended in PBS buffer with concentration of 30%Hct, then the stiffened RBCs (0.02%v/v) were added in to form the 30%Hct sample, and the storage modulus measurement was provided in Supplementary Materials V.

*PBS sample:* stiffened RBCs suspended in PBS buffer with concentration of 0.02%v/v.

*PVP sample:* polyvinyl pyrrolidone (PVP, Mw = 360 000, Sigma Aldrich) suspended in PBS buffer with concentration of 7.5%wt, and then the stiffened RBCs (0.02%v/v) were added in to compose the PVP sample.

### Data Availability

The datasets generated during and/or analyzed during the current study are available from the corresponding author on reasonable request.

## Electronic supplementary material

supplementary video-movie 1

supplementary video-movie 2

supplementary information
