# Supplementary material for: Margination of Stiffened Red Blood Cells Regulated By Vessel Geometry
Source: Sci Rep. 2017 Nov 10;7:15253. doi: 10.1038/s41598-017-15524-0 (PMC5681636; doi:10.1038/s41598-017-15524-0)
Supplement: Supplementary file 3 — supplementary information [file 41598_2017_15524_MOESM3_ESM.pdf]

Supporting Information

Margination of Stiffened Red Blood Cells Regulated By Vessel Geometry

Yuanyuan Chen[1], Donghai Li[1], Yongjian Li[1], Jiandi Wan[2], Jiang Li[3], Haosheng Chen[1,*]

I: RBCs flowing observation *in vivo*.

The distribution data of RBCs flowing in blood vessel *in vivo* were collected from 6mice as two groups, each mice contributed 200 RBCs to the statistical results. The RBCs flowing observations of each mice were shown in Fig. S1(a1) ~ (b3), and the corresponding videos are provided in *Supplementary Materials (IV)-videos*. All the videos were captured under X10 objective using a high speed camera (M110, Phantom Co.). Furthermore, Fig. 1 in the main article showed the slice observation of mice ear from the stiffened RBCs experiment group, here Fig. S1(c1) ~ (c3) showed the slice results from the control group, which were injected into normal RBCs in the flowing observation experiment.

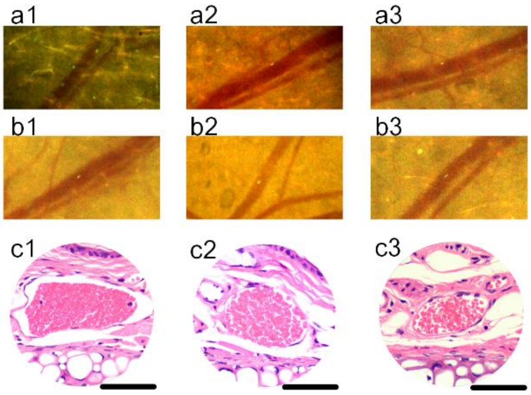

Figure S1. (a1) ~ (a3) show the views of stiffened RBCs flowing observation in mice ear vessel as experiment group. (b1) ~ (b3) show the views of normal RBCs flowing observation as control group. (c1) ~ (c3) show the slice observation of mice ear from control group, and the scale bar is 100μm.

II: Margination of stiffened RBCs in rectangular channel with height of 40μm .

Marginaiton of stiffened RBCs was performed in rectangular channel with height of 40μm. The viscoelasticity of PVP solution is not so strong as that of RBCs suspension, as Fig.S4 proved that,

margination did not happen in this rectangular with the length of 3cm. Thus we lower the height of the channel in Fig.4(b) to see the margination of the stiffened cells on both the top and bottom wall of the channel to show a more obvious margination effect compared to that in the PBS solution.

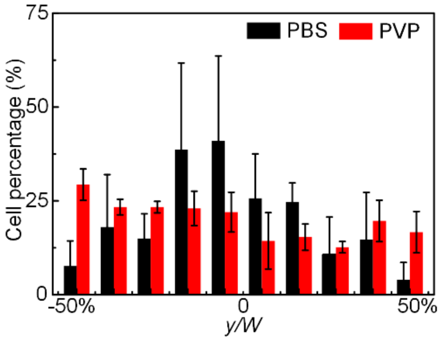

Figure S2. The experiment results of margination in 40μm height rectangular channel in PBS and PVP solution.

III: AFM-based measurement of RBC's modulus.

The stiffness of RBCs are evaluated by Young's modulus and measured on an AFM, as the schematics showed in Fig. S2(a). The normal stiffened RBCs were immersed in PBS solution and squeezed by a SiO_2 microsphere on the tip of the AFM probe, and then the modulus of the squeezed RBC was acquired according to their deformability under the pressure. 100 RBCs were measured, and their modules have been shown in Fig. S2(b). It is obvious that the stiffened RBCs have a higher Young's modulus than normal RBCs, therefore, the stiffened RBCs would have different margination behaviors from normal RBCs.

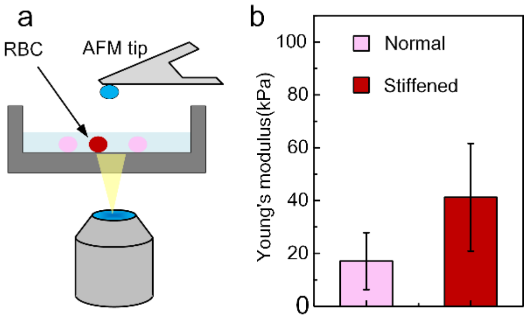

Figure S3. Young's modulus measurement of RBC. (a) Schematics of auto force microscope based Young's modulus measurement. (b) Young's modulus of normal RBC and stiffened RBC.

IV: Numerical calculation of the normal stress on the cross section of the channel.

The geometry model was established in Comsol Multiphysics software according to the experimental conditions. Here, the shear rate and normal stress distribution in the geometries of triangular, circular and rectangular are demonstrated in Fig. S3. The bottom width of triangle is 100μm, the diameter of the circular channel is 100μm, and the size of the rectangular channel is

40μm×100μm (height×width)for the channel shape controlled experiment, respectively. Therefore, laminar flow models were applied in all of the simulations. Under the same flow rate, the stream wise velocity field for the viscoelastic flow is the same as that for the Newtonian flow. Equations of mass conservation and Navier-Stokes equations were solved numerically with the commercial software - Comsol Multiphysics. About the boundary condition, the initial inlet velocity is set as 1mm/s and the outlet is free. To guarantee that the calculation results are mesh independent, results obtained by meshes with different grid densities had been compared. Meshes with a degree of freedom higher than 10^6 were applied finally, which had been checked to be fine enough to gain the stable results for the present simulations.The meshing process were done in Comsol Multiphysics software using the fine pattern, and the results are shown in Fig. S3. The algorithm was set as Newton's method according to Comsol, and the criterion of iterative convergence in these simulations was 10^{-4}.

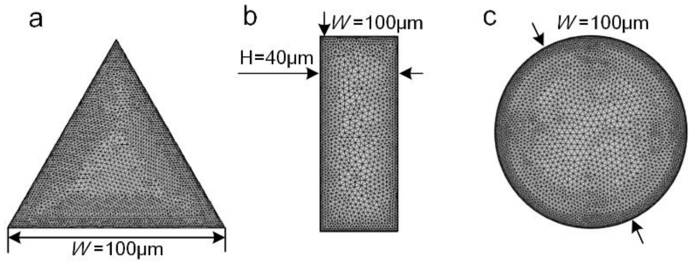

Figure S4. (a) The meshing result of triangular circular channel in Comsol. (b)The meshing result of circular channel in Comsol. (c) The meshing result of rectangular channel in Comsol.

V: Storage modulus measurement of 30%Hct RBCs suspension.

The viscoelasticity of the 30%Hct RBCs suspension were expressed by storage modulus (G') and measured on a rotation rheometer (MCR302, Anton paar, Co.) with a cone-plate geometry (49.981mm in diameter, 0.993° cone angle and 0.1mm gap) at the shear mode. The shear rate was set from 10Hz to 0.1Hz, and the results was shown in Fig. S5.

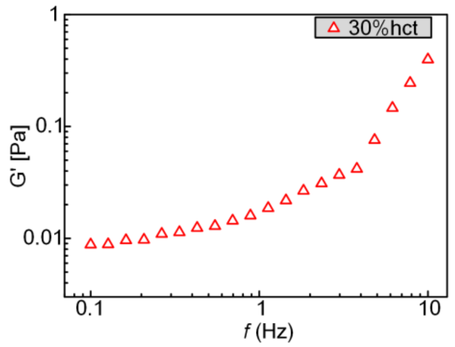

Figure S5. The storage modulus (G') measurement of 30%Hct RBCs suspension.
